# CPEB1 Controls NRF2 Proteostasis and Ferroptosis Susceptibility in Pancreatic Cancer

**DOI:** 10.7150/ijbs.95962

**Published:** 2024-06-03

**Authors:** Shuxia Zhang, Jingnan Huang, Zhangzhang Lan, Yanlin Xiao, Youyou Liao, Shiva Basnet, Piying Huang, Yunze Li, Jingyu Yan, Yuling Sheng, Wenwen Zhou, Qi Liu, Haoyuan Tan, Yi Tan, Leyong Yuan, Lisheng Wang, Lingyun Dai, Wenyong Zhang, Changzheng Du

**Affiliations:** 1 Key University Laboratory of Metabolism and Health of Guangdong, Biochemistry Department, School of Medicine, Southern University of Science and Technology, 1088 Xueyuan Avenue, Shenzhen, Guangdong 518055, P.R. China.; 2 Department of Gastroenterology, The First Affiliated Hospital (Shenzhen People's Hospital), Southern University of Science and Technology, 1017 Dongmen North Road, Shenzhen, Guangdong 518020, P.R. China.; 3 Department of Geriatrics, and Shenzhen Clinical Research Centre for Geriatrics, The First Affiliated Hospital (Shenzhen People's Hospital), Southern University of Science and Technology, 1017 Dongmen North Road, Shenzhen, Guangdong 518020, P.R. China.; 4 School of Medicine, Southern University of Science and Technology, 1088 Xueyuan Avenue, Shenzhen, Guangdong 518055, P.R. China.; 5 Clinical laboratory, Southern University of Science and Technology Hospital, 6019 Liuxian Street, Xili Avenue, Shenzhen, Guangdong 518055, P.R. China.; 6 Beijing Tsinghua Changgung Hospital & Tsinghua University School of Medicine, 168 Litang Road, Changping District, Beijing 102218, P.R. China.

**Keywords:** CPEB1, p62/SQSTM1, NRF2, pancreatic cancer, ferroptosis

## Abstract

Pancreatic cancer is the deadliest malignancy with a poor response to chemotherapy but is potentially indicated for ferroptosis therapy. Here we identified that cytoplasmic polyadenylation element binding protein 1 (CPEB1) regulates NRF2 proteostasis and susceptibility to ferroptosis in pancreatic ductal adenocarcinoma (PDAC). We found that CPEB1 deficiency in cancer cells promotes the translation of p62/SQSTM1 by facilitating mRNA polyadenylation. Consequently, upregulated p62 enhances NRF2 stability by sequestering KEAP1, an E3 ligase for proteasomal degradation of NRF2, leading to the transcriptional activation of anti-ferroptosis genes. In support of the critical role of this signaling cascade in cancer therapy, CPEB1-deficient pancreatic cancer cells display higher resistance to ferroptosis-inducing agents than their CPEB1-normal counterparts *in vitro* and *in vivo*. Furthermore, based on the pathological evaluation of tissue specimens from 90 PDAC patients, we established that CPEB1 is an independent prognosticator whose expression level is closely associated with clinical therapeutic outcomes in PDAC. These findings identify the role of CPEB1 as a key ferroptosis regulator and a potential prognosticator in pancreatic cancer.

## Introduction

Pancreatic cancer is a leading cause of cancer death worldwide, with a 5-year survival rate of approximately 10% 1, 2. Over the past decade, advances in diagnosis, surgery, chemotherapy, radiotherapy, and immunotherapy have made relevant but only modest incremental improvements in the therapeutic outcomes of pancreatic cancer 2. Owing to the poor response to chemotherapy, surgery remains the only way to cure the disease at an early stage 2, 3, while late-stage pancreatic cancer remains a formidable challenge for oncologists.

Ferroptosis, an iron-dependent form of regulated cell death driven by iron oxidation and lipid hydroperoxide accumulation, is increasingly being recognized as a promising strategy in cancer therapy 4, 5. Both experimental reagents (such as erastin and RSL3) and FDA-approved drugs (such as sorafenib, sulfasalazine and statins) exhibit tumor-suppressive effects by inducing ferroptosis in cancer cells 5. However, susceptibility to ferroptosis is related to numerous biological processes. It is known that the metabolism of iron and polyunsaturated fatty acids, the biosynthesis of glutathione and other molecules in the oxidation-reduction reaction, and the breaking and repair of the cell membrane, together with autophagy, form a complicated network controlling ferroptosis susceptibility 4, 6. Due to the complexity of ferroptosis, which cancer phenotype is sensitive to and can benefit from ferroptosis therapy remains an important clinical question to be answered.

During our study of ferroptosis in pancreatic cancer, we found that cytoplasmic polyadenylation element binding protein 1 (CPEB1) plays a critical role in controlling susceptibility to ferroptosis and is closely associated with clinical therapeutic outcomes. CPEB1 is a sequence-specific mRNA-binding protein that controls the polyadenylation elongation process of mRNA 7-9. For the target mRNA with cytoplasmic polyadenylation elements (CPEs), CPEB1 exerts its translational regulatory function either by controlling the elongation of poly(A) with other translational regulators, such as poly(A) polymerases GLD-2 or GLD-4 7, or by regulating the splicing of the 3'-untranslated region (UTR) of the mRNA in coordination with splicing factors, such as U2AF65 9, to accelerate or block protein translation. CPEB1 tends to play a tumor suppressive role in cancer: it is commonly low-expressed in solid tumors through DNA hypermethylation 10, 11; moreover, it inhibits tumor growth and metastasis, and attenuates stemness in multiple cancer types including breast cancer 12, glioblastoma 13, gastric cancer 10, colorectal cancer 11 and hepatic cancer 14. However, the involvement of CPEB1 in pancreatic cancer and its role in cancer therapy remains unclear.

In the present study, we explored the role and underlying mechanism of CPEB1 in the regulation of ferroptosis in pancreatic cancer. We revealed that CPEB1 deficiency enhances proteostasis of nuclear factor erythroid 2-related factor 2 (NRF2, coding gene: *NFE2L2*) by promoting the translation of p62 (coding gene: *SQSTM1*). We also identify CPEB1 as a potential prognosticator of ferroptosis therapy in pancreatic cancer.

## Materials and Methods

### Cell lines and cell culture

All human cancer cell lines were purchased from the American Type Culture Collection (ATCC) or European Collection of Authenticated Cell Cultures (ECACC) except JF-305, which was a gift from Prof. Jing Gao. The pancreatic cancer cell line PANC-1 was cultured in Dulbecco's Modified Eagle's Medium (DMEM) (Gibco, Cat# 11995-065) supplemented with 10% fetal bovine serum (FBS; Corning, Cat# 35-010-CV) and 1% penicillin/streptomycin (Gibco, Cat #15140-122); while JF-305 and AsPC-1 were cultured in Roswell Park Memorial Institute (RPMI) 1640 medium supplemented with 10% FBS and 1% penicillin/streptomycin. The colorectal cancer cell line HCT116 was cultured in McCoy's 5A medium and HT29 in RPMI 1640 medium with 10% FBS, as previously described. U-251MG human glioblastoma cells were cultured in DMEM supplemented with 10% FBS and 1% penicillin/streptomycin. Gastric cancer cell lines MGC-803 and HGC-27 were cultured in RPMI 1640 medium with 10% FBS and 1% penicillin/streptomycin. All the cell lines were cultured in a humidified atmosphere at 37°C and 5% CO_2_.

### Cell viability and proliferation assays

Cell viability following treatment with ferroptosis inducers was evaluated using the Cell Counting kit-8 (CCK-8, Beyotime, Cat# C0040), according to the manufacturer's protocol. Briefly, cells were seeded in 96-well plates at a density of 1×10^4^ cells/well. After culturing for 12 hours, cells were treated with erastin (MCE, Cat# HY-15763) or RSL3 (MCE, Cat# HY-100218A) for 72 hours. CCK-8 solution was then added to each well at a concentration of 10% and the cells were incubated at 37°C for 1-2 hours before measurement. The absorbance of each well was measured at 450 nm using a Synergy HTX microplate reader (Agilent, USA).

Cell proliferation was evaluated using a colony formation assay. Briefly, cells plated at a confluence of 70%-80% were treated with ferroptosis inducers for 24 hours, then seeded on 6-well plates at a density of 800 cells per well with fresh medium, growing for another 14 days to form colonies. The colonies were fixed with paraformaldehyde (4.0% v/v), stained with crystal violet (0.5% w/v), and counted manually. Student's *t*-test was performed to analyze the differences between the groups.

### RNA extraction, reverse transcription and quantitative real-time PCR (RT-qPCR)

Total RNA from cells was isolated using an RNA extraction kit (Vazyme, Cat# RC112-01) following the manufacturer's instructions. Complementary DNA (cDNA) was reverse-transcribed from 1 μg of total RNA using a Reverse Transcriptase Kit (Vazyme, Cat# R312-02). Real-time quantitative PCR was performed on a QuantStudio 7 Flex system (Thermo fisher, USA) with a SYBR Green PCR Kit (TransGen, Cat#AQ132-21). Primer information for the target genes is shown Supplementary List 1. The expression level of *B2M* served as an internal control for the normalization of target gene expression. All RT-qPCR was performed three times independently, as shown with the representative results.

### Protein extraction, immunoblots and immunoprecipitation assay

Total protein was extracted from cells using lysis buffer containing 1 mM DTT and 1% sodium dodecyl sulfate (SDS), as previously described 15. Immunoblots was performed as described previously 16. In brief, proteins were separated on 8-12% SDS-PAGE gels and transferred onto polyvinylidene fluoride (PVDF) membranes (Millipore, Cat# IPFL00010) or nitrocellulose (NC) membranes (Amershan protran, Cat# 10600002), which were incubated overnight with specific antibodies against target proteins at 4°C. Blots were probed with horseradish peroxidase-conjugated secondary antibodies (ZSGB-Bio company, Cat# ZB-2301, ZB-2305), followed by detection with enhanced chemiluminescence (ECL, Advansta, Cat# K-12045-D50) by ChampChemi 610 Plus System (SAGE, China). For immunoprecipitation, cells were lysed in p300-lysis buffer as previously reported 16. The protein extracts were incubated with anti-FLAG M2 agarose beads (Sigma, Cat# A2220), or mouse IgG plus A/G agarose beads (Thermo Fisher, Cat# 20421) at 4°C for 4-6 hours. The immunoprecipitants were washed three times on ice with precooled lysis buffer and eluted with SDS loading buffer by boiling for 10 minutes before being analyzed by immunoblotting.

### Polysome profiling assay

Polysome profiling assay was performed as previously described 17. PANC-1 derivative cells (Control or CPEB1-ko) were harvested and lysed on ice with Polysome lysis buffer (20 mM Tris HCl pH 7.4, 5 mM MgCl_2_, 100 mM NaCl, 100 μg/mL cycloheximide, 1% Triton X-100, 40 U/mL RNasin, and protease inhibitor cocktail), followed by centrifugation at 12000×g at 4°C. Clear supernatants from lysates were transferred into 10%-50% sucrose gradients (sucrose diluted in a buffer composed of 25 mM Tris HCl pH 7.4, 5 mM MgCl_2_ and 100 mM NaCl) and centrifuged at 35,000 rpm (SW41Ti Beckman Rotor) for 3 hours at 4 °C. Absorbance at 260 nm was monitored to record the polysome profiling curve using a Piston Gradient Fractionator (Biocomp, USA). Polysomal RNA was extracted as described above, used for the RT-qPCR assay.

### Ribonucleoprotein immunoprecipitation (RIP) assay

The RIP assay was performed as previously described 18. Briefly, Cells overexpressing FLAG-tagged CPEB1 were harvested and crosslinked with 1% (v/v) formaldehyde for 10 minutes at room temperature. Cells were then lysed on ice with IP buffer (20 mM Tris-HCl, pH 8.0, 200 mM NaCl, 1 mM EDTA, 1 mM EGTA, 0.5% Triton X-100, 0.4 U/μl RNasin and protease inhibitor cocktail), followed by immunoprecipitation with anti-FLAG M2 agarose beads (Sigma, Cat# A2220) or mouse IgG plus A/G agarose beads (Thermo Fisher, Cat# 20421) at 4°C for 4 hours. The beads were washed three times and incubated with Proteinase K for decrosslinking. RNA was extracted from the samples using TRIzol (TransGen, Cat# ET111-01-V2).

### Poly(A) tail length assay

The poly (A) tail length assay was performed using the extension Poly(A) Test (ePAT) method, as previously described 19. Briefly, total RNA extracted from cells was mixed with a PAT-anchor primer for annealing, incubated at 80°C for 5 min, and cooled to room temperature. The RNA with Poly(A) was tagged with an anchor sequence on its 3' terminus by using Klenow polymerase (NEB, Cat# M0210V) with the reaction buffer (5 mM DTT, 0.5 mM dNTPs, 40U RNase inhibitor (Vazyme, Cat# R301-02) and 1xSuperscript IV mixture (Life Technologies, Cat# 18090200)), and incubated at 25°C for 1 hour for the RNA extension reaction. The 3'-tagged adenylated RNA was then reverse-transcribed into cDNA for further PCR amplification and Sanger sequencing. The primer sequences are listed in Supplementary List 2.

### Reactive oxygen species (ROS) assay, glutathione (GSH)/glutathione disulfide (GSSG) assay, malondialdehyde (MDA) assay and lactate dehydrogenase (LDH) assay

The intracellular ROS level was detected using 2',7'-dichlorodihydrofluorescein diacetate (DCFH-DA) fluorescent probe (Beyotime, Cat# S0033S) following the manufacturer's protocol. GSH and GSSG were detected using a GSSG/GSH Quantification Kit (Dojindo, Cat# G263) following the manufacturer's protocol, and the results were normalized to the protein concentration measured using the BCA assay kit (Beyotime, Cat# P0012S). Similarly, intracellular MDA was detected using an MDA assay kit (TBA method; Dojindo, Cat# M496) following the manufacturer's protocol. The LDH in the cell culture medium was detected using an LDH Cytotoxicity Assay Kit (Beyotime, Cat# C0016) following the manufacturer's protocol. The cytotoxicity index was calculated using the formula 20: (test LDH release-spontaneous release)/maximal release.

### Proteomic analysis

The cell pellets were lysed with 200 μL lysis buffer (1% sodium deoxycholate (SDC, Sigma, Cat# D6750), 8 M urea, 100 mM triethylammonium bicarbonate (TEAB, Sigma, Cat# T7408) and 1x protease inhibitor). After reduction with 20 mM dithiothreitol (DTT, Sigma, Cat# D9779) and alkylation with 40 mM iodoacetamide (IAA, Sigma, Cat# I1149), proteins were digested with trypsin (Promega, Cat# V5111) at an enzyme: protein as 1:50.

LC-MS/MS analysis was performed using an EASY-nLC 1200 system (Thermo Fisher, USA) coupled to an Orbitrap Eclipse Tribrid mass spectrometer (Thermo Fisher, USA). A data-independent acquisition (DIA) approach was applied to obtain quantitative protein information. Raw files were analyzed using DIA-NN version 1.8.1 21 against the human database downloaded from SwissProt (20221212, 20401 entries), and differential protein expression was analyzed with the DEP R package (1.20.0) 22. Pathway enrichment and functional analysis was performed using Metascape 23 and the clusterProfiler package (4.4.4) in R programming (4.2.2).

### Immunofluorescence (IF) assay

Cells cultured in chambered coverslip (Ibidi, Cat# 80826) were fixed in 4% (v/v) paraformaldehyde (30 minutes at room temperature), washed with PBS, and blocked with 5% (v/v) bovine serum albumin (BSA) for 1 hour. Subsequently, the cells were incubated overnight at 4˚C with the anti-NRF2 antibody (1:400, Proteintech, Cat#16396-1-AP). After washing with PBS, the fluorescein coupled secondary antibody (1:400, Huabio, Cat# HA1121) was added and incubated for 1 hour at 37˚C in a humidity chamber. Finally, DAPI (4',6-diamidino-2-phenylindole) was utilized to stain the nuclei (100ng/ml, 10 minutes in room temperature). The samples were then scanned using a confocal microscope (Zeiss LSM980, SUSTech Core Research Facilities).

### Compounds, plasmids and antibodies

All compounds, small-molecule inhibitors, antibodies, oligonucleotides and plasmids used in this study are summarized in Supplementary List 3-6. Cell lines with *CPEB1* knockout were constructed using the pX459 vector (Addgene #62988) with two sgRNAs. All lentiviruses were packaged using 293FT cells (Invitrogen, Cat# R700-07), with a MOI value of 5.

### Mouse xenografts and treatments

Eight-week-old female nude mice were obtained from, and maintained at, the SUSTech Laboratory Animal Center. All mice were randomly divided into four groups (Ctrl+Vehicle group, n=6; CPEB1-ko+Vehicle group, n=5; Ctrl+Erastin group, n=5; CPEB1-ko+Erastin group, n=6). Tumor implantation was performed with 2×10^6^ pancreatic cancer cells injected into the subcutaneous layer on one side of the flank. Tumor size was measured twice a week, and tumor volume was calculated using the formula: length × width^2^× 0.5. Mice were euthanized when the long diameter exceeding 1.5 cm or the tumor volume reached 1500 mm^3^. Mice were intraperitoneally administered erastin (30 mg/kg) or vehicle control (corn oil) when the tumor volume reached 100 mm^3^. The treatment was administered three times per week for 2 weeks. The observation duration lasted for two months (60 days) since the first treatment, and all mice were euthanized at the end-point regardless of tumor size.

### Patients, tissue microarray and immunohistochemistry (IHC)

Formalin-fixed, paraffin-embedded human pancreatic cancer and tumor-adjuvant tissue microarray slides (Cat# HPanA170Su04) were purchased from Outdo Biotech (Shanghai, China). The slide contained tissue samples from 90 individual cases obtained from and authorized by the National Human Genetic Resources Sharing Service Platform (Platform No: 2005DKA21300).

The expression levels of CPEB1, p62 and NRF2 were assessed by IHC staining of pancreatic cancer and tumor-adjacent tissues. The immunoreactive score (IRS) was calculated as previously reported 24. Protein expression was classified into “low-expression” and “high-expression” based on a cut-off score of 6, namely low-expression is defined as IRS<6, while high-expression is defined as IRS≥6, as previously reported 24. All slides were read by two senior pathologists who were blinded to clinical data. In the discrepant case, both pathologists reached a consensus before the final scores were determined.

### Statistical analysis

All experiments were independently repeated at least three times. Quantitative data are presented as mean ± s.d., and were compared using *Student's t*-test if the data followed a normal distribution; otherwise, the data were compared using a nonparametric test. Categorical variables were analyzed using the chi-squared test. The relationship between variables was analyzed using Pearson correlation analysis. Survival was assessed using the Kaplan-Meier method with a log-rank test. Data were analyzed using SPSS software (version 19.0; SPSS Inc., USA). All tests were two-sided, and a *P* value less than 0.05 was deemed statistically significant.

## Results

### CPEB1 deficiency increases cellular resistance to ferroptosis in pancreatic cancer

As CPEB1 is involved in cancer progression and therapeutic resistance 12, 14, we wondered whether its expression level is altered in the majority of cancer types. By analyzing the expression level of CPEB1 among the 31 cancer types in The Cancer Genome Atlas (TCGA) database, we found that there are 21 cancer types in which CPEB1 is remarkably less expressed than in the matched tumor-adjacent tissues (Figure S1A), which is consistent with previous reports that *CPEB1* is hypermethylated and expressed at low levels in solid tumors 10, 11. In pancreatic cancer, low-expression of CPEB1 is associated with later TNM stage (Figure S1B), and potentially worse survival (Figure S1C, S2). These data prompted us to further study the oncological significance of CPEB1 deficiency in pancreatic cancer.

**Figure 1:**
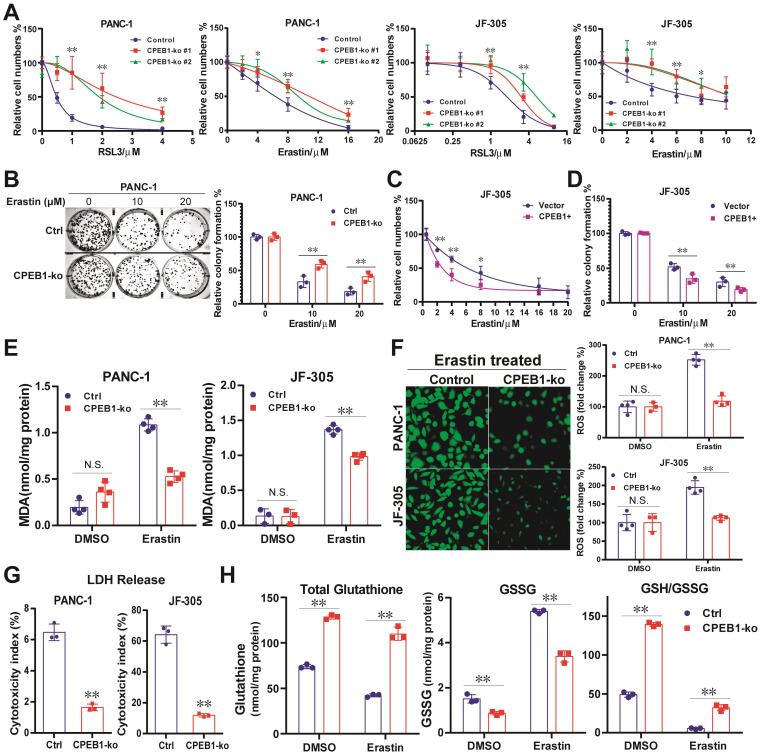
** CPEB1 deficiency increases ferroptosis resistance in pancreatic cancer. (A)**. Isogenic cell lines derived from PANC-1 and JF-305 (Control or CPEB1-ko) were treated with ferroptosis inducers including RSL3 and erastin for 72 hours, respectively; and cell numbers were determined by CCK-8 assay. **(B)**. PANC-1 derivative cell lines without (Control, Ctrl) or with CPEB1 knockout (CPEB1-ko) were treated with indicated doses of erastin for 24 hours, and colony formation assays were performed with 800/well to determine the proliferation ability of treated cells. **(C)**. JF-305 cells without (Vector) or with exogenous CPEB1 overexpression (CPEB1+) were treated with indicated doses of erastin for 72 hours, and cell numbers were determined by CCK-8 assay. **(D)**. JF-305 cells without (Vector) or with exogenous CPEB1 overexpression (CPEB1+) were treated with indicated doses of erastin for 24 hours, and colony formation assays were performed (800/well) to determine the proliferation ability. **(E-G)**. Derivatives of PANC-1 and JF-305 (Control or CPEB1-ko) were treated with erastin of 10 μM for 12 hours, MDA (E) was detected using TBA method; ROS (F) was detected using DCFH-DA fluorescent probe by both fluorescence imagine (left) and microplate reader (right); and LDH (G) was detected using the LDH Cytotoxicity Assay. **(H)**. PANC-1 derivative cell lines (Control or CPEB1-ko) were treated with erastin of 10 μM for 12 hours, total glutathione and glutathione disulfide (GSSG) were detected using GSSG/GSH Quantification Kit, normalized with protein concentration; and reduced glutathione was calculated to evaluate the GSH/GSSG ratio. *P < 0.05; **P < 0.01. N.S., no significance.

To mimic CPEB1 deficiency in tumors, we constructed CPEB1-knockout (CPEB1-ko) pancreatic cancer cell lines derived from PANC-1 and JF-305 cells (Figure S3). These CPEB1-deficient cancer cells exhibited remarkably higher resistance to various ferroptosis inducers than their CPEB1-normal counterparts, as identified by CCK-8 and colony formation assays (Figure 1A-B). Complementarily, overexpression of CPEB1 sensitized cancer cells to ferroptosis inducers (Figure 1C-D). The biochemical markers of ferroptosis, including the production of lipid peroxidation (MDA) and ROS, the LDH reflecting membrane damage, and the ratio of GSH/GSSG as a representative marker of antioxidant capacity, were also measured as shown in Figure 1E-H, clearly supporting that CPEB1 deficiency increases ferroptosis resistance in pancreatic cancer.

### CPEB1 deficiency enhances the NRF2 proteostasis in pancreatic cancer

**Figure 2:**
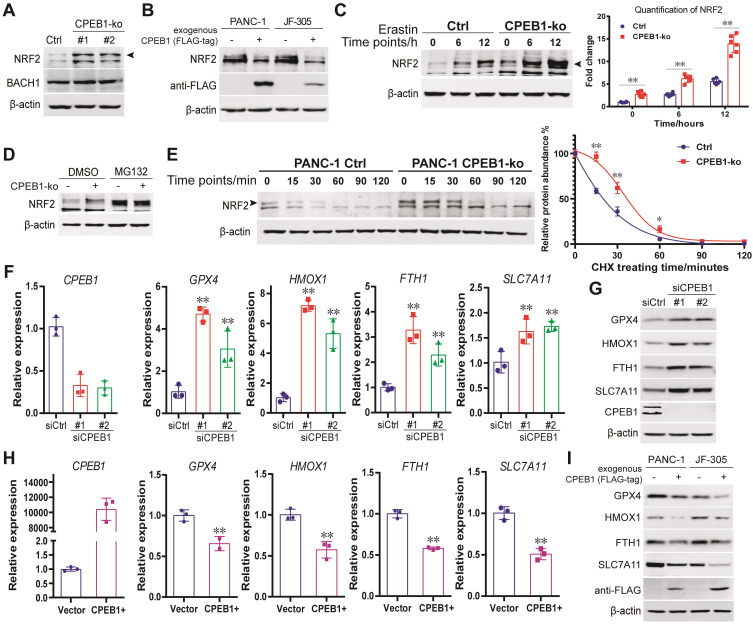
** CPEB1 deficiency enhances the NRF2 proteostasis in pancreatic cancer (A)**. The abundance of NRF2 (as arrow shows) and BACH1 were detected in PANC-1 Control (Ctrl) and its matched CPEB1-ko derivatives by immunoblotting. **(B)**. The abundance of NRF2 without or with exogenous FLAG-tagged CPEB1 overexpression in PANC-1 and JF-305 cells was detected by immunoblotting. **(C)**. PANC-1 derivative cell lines (Control or CPEB1-ko) were treated with erastin of 10 μM for indicated time points, and the abundance of NRF2 (as arrow shows) was determined by immunoblotting (as arrow shows) (left panel) with quantification (right panel). **(D)**. PANC-1 derivative cell lines (Control or CPEB1-ko) were treated with DMSO or MG132 (10 μM for 12 hours), and the abundance of NRF2 was determined by immunoblotting. **(E)**. PANC-1 derivative cell lines (Control or CPEB1-ko) were treated with cycloheximide for indicated time points, and the abundance of NRF2 (as arrow shows) was examined by immunoblotting (left panel). The quantification of relative abundance of NRF2 compared to the starting time point is calculated and shown in the right panel. **(F)**. Total RNA from PANC-1 cells, without (siCtrl) or with CPEB1 knockdown (siCPEB1) were used for RT-qPCR analysis using indicated primers. **(G)**. Protein lysates from PANC-1 cells, without (siCtrl) or with CPEB1 knockdown (siCPEB1), were used for immunoblots using indicated antibodies. **(H)**. Total RNA from PANC-1 cells, without (Vector) or with CPEB1 overexpression (CPEB1+) were used for RT-qPCR assay using indicated primers. **(I)**. Protein lysates from PANC-1 and JF-305 cells, without or with CPEB1 overexpression (FLAG-tagged), were used for immunoblots using indicated antibodies.

So far, the only ferroptosis regulator reported to be under the control of CPEB1 is TWIST1, an inhibitor of ATF4 25. However, TWIST1 is hardly expressed in pancreatic cancer cells (Figure S4). Instead, we found that NRF2, another potent regulator of ferroptosis 26, 27, mediates the ferroptosis downstream of CPEB1 in pancreatic cancer based on the following findings: First, we detected the protein abundance of NRF2 (a key transcription factor in response to oxidative stress) and BACH1 (a transcriptional repressor antagonistic to NRF2 28) in the CPEB1-ko and -normal cells by immunoblotting, and found that CPEB1 loss remarkably upregulated NRF2 without affecting BACH1 (Figure 2A), whereas overexpression of CPEB1 complementarily downregulated NRF2 (Figure 2B). Second, CPEB1-deficient PANC-1 cells showed enhanced NRF2 upregulation upon treatment with the ferroptosis inducer erastin compared to their CPEB1-normal counterparts (Figure 2C), in agreement with the scenario of ferroptosis resistance induced by CPEB1 loss. Third, CPEB1 loss-induced NRF2 upregulation was not associated with a higher level of NRF2 transcript (Figure S5A) and was rescued by the proteasome inhibitor MG132 (Figure 2D). Furthermore, a protein stability assay revealed a retarded rate of NRF2 degradation in CPEB1-ko cells (Figure 2E), suggesting that CPEB1 loss improves the stability of NRF2. Finally, as a transcription factor that induces ferroptosis resistance, NRF2 promotes the transcription of numerous anti-ferroptosis genes, including* GPX4, HMOX1*, and *SLC7A11*, as validated by reverse transcription and RT-qPCR assays (Figure S5B). We then demonstrated that loss of CPEB1 in pancreatic cancer cells activated the expression of NRF2-target genes (Figure 2F-G); and that overexpression of CPEB1 complementarily downregulated the expression of these genes (Figure 2H-I). As the nuclear localization of NRF2 is crucial for its transcriptional activity 29, we conducted two independent experiments to determine the impact of CPEB1 on the subcellular localization of NRF2: neither the immunoblotting data with fractionalized protein (cytoplasmic and nuclear fraction) (Figure S5C) nor the immunofluorescence result (Figure S5D) supported CPEB1 inducing NRF2 translocation. All the data above suggest CPEB1-loss enhances the stability of NRF2 without affecting its expression or translocation, and thereby activates the transcription of anti-ferroptosis genes.

### CPEB1 deficiency promotes p62 translation by facilitating mRNA polyadenylation

**Figure 3:**
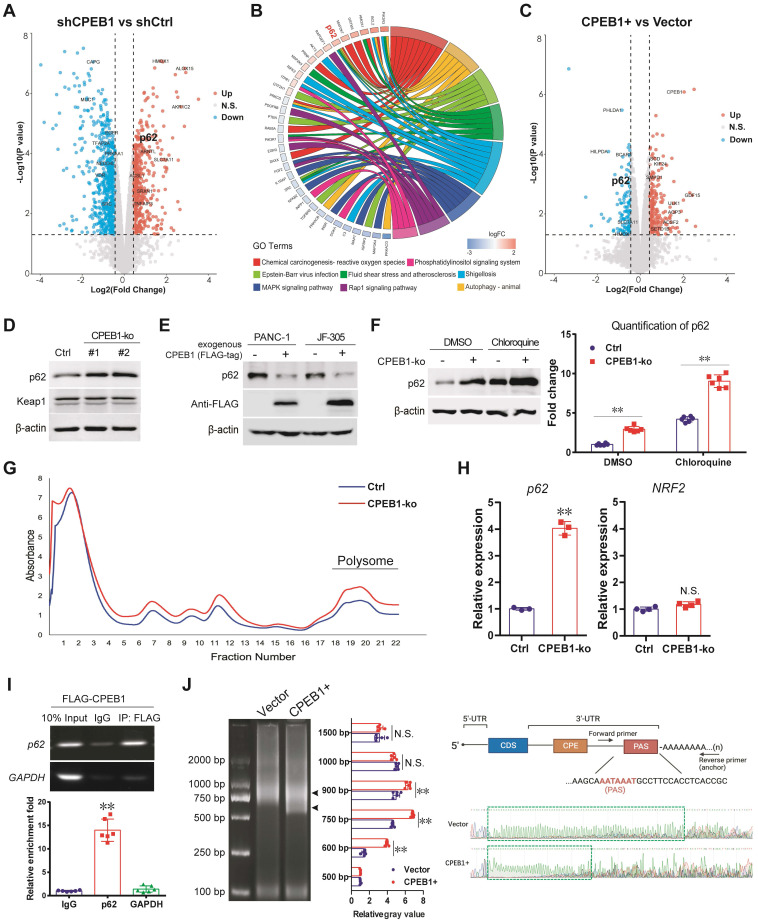
** CPEB1 deficiency promotes p62 translation by facilitating mRNA polyadenylation. (A-C)** Proteomic analysis using JF-305 derivative cell lines without (shCtrl) or with CPEB1-knockdown (shCPEB1) (A-B), or without (Vector) or with CPEB1 overexpression (CPEB1+) (C). Volcano plot (A) showed differentially expressed proteins (DEPs) between shCPEB1 and shCtrl. GO chord plot (B) displayed GO analysis for the DEPs, linking the DEP with biological function. Volcano plot (C) showed DEPs between CPEB1-overexpression and Control groups (CPEB1+ vs Vector), using the same method as described in (B). **(D)**. The abundance of p62 and KEAP1 were detected in PANC-1 Control and its matched CPEB1-ko derivatives. **(E)**. The abundance of p62 without or with exogenous FLAG-tagged CPEB1 overexpression in PANC-1 and JF-305 cells was determined by immunoblotting. **(F)**. JF-305 derivative cell lines (Control or CPEB1-ko) were treated with DMSO or Chloroquine of 20 μM for 12 hours, the abundance of p62 was determined by immunoblotting (left panel). The quantification of the p62 abundance is shown in the right panel. **(G-H)**. Polysome profiling analysis was performed in PANC-1 derivative cell lines (Control or CPEB1-ko) (G), and RNA in the polysome fraction (as labeled) was collected and reversely transcripted. (H). The mRNA abundance of p62 and NRF2 in the polysome fraction was detected by RT-qPCR assay (right panel). **(I)**. RIP assay was performed in the PANC-1 cells expressing exogenous FLAG-tagged CPEB1 (FLAG-CPEB1) to determine the interaction between CPEB1 and the mRNA of p62. The enrichment of mRNA was quantified with IgG normalization, shown in the bottom panel. **(J)**. Poly(A) tail assay was performed in PANC-1 cells without (Vector) or with CPEB1 overexpression (CPEB1+) (left panel, arrows show the bands of Poly(A) fractions), with quantitative analysis (middle panel); followed by Sanger sequencing of the Poly(A) in 3'-UTR (right panel, labeled with green frame). The primers designed around PAS element was shown in the right-upper panel. *P < 0.05; **P < 0.01; N.S., no significance.

To gain further insight into the effect of CPEB1 loss on NRF2 proteostasis, we performed a proteomic analysis using JF-305 derivative cells with or without CPEB1 knockdown (Figure 3A). Among the differentially expressed proteins (DEPs), we noticed that p62, which is involved in oxidative stress and proteostasis regulation, was significantly upregulated upon CPEB1 loss (Figure 3A-B). As previous reports have shown that p62 maintains NRF2 stability by sequestering the E3 ligase KEAP1 from NRF2 30, 31, we hypothesized that p62 is a potential mediator of CPEB1 loss-induced NRF2 upregulation. We noted that the proteomic data from CPEB1-overexpressing cells also provided additional evidence that p62 was downregulated upon CPEB1 overexpression (Figure 3C). We then investigated the regulatory mechanism between CPEB1 and p62 by performing the following experiments: First, we confirmed by immunoblotting that knockout of CPEB1 resulted in a remarkable increase in p62 abundance without affecting KEAP1 (Figure 3D), whereas overexpression of CPEB1 complementarily induced a decrease in p62 abundance (Figure 3E), consistent with the proteomic data. Notably, this p62 up- or down-regulation induced by CPEB1 loss or overexpression was not associated with transcriptional regulation, determined by RT-qPCR assay (Figure S6). Since p62 abundance is highly associated with autophagy activity 32, we inhibited cellular autophagy using chloroquine, a commonly used lysosomal inhibitor, and found that it did not block the p62 upregulation induced by CPEB1 loss (Figure 3F), thus excluding autophagy-induced p62 alteration. Second, the polysome profiling assay revealed a significantly activated translation induced by CPEB1 loss (Figure 3G), and an improved proportion of *p62* mRNA in the polysome fraction in CPEB1-deficient cells (Figure 3H), supporting the regulatory mechanism by which loss of CPEB1 promotes the translation of p62, whereas the polysomal RNA of NRF2 showed no change in the CPEB1-deficient cells (Figure 3H). Third, RIP assay showed that CPEB1 directly binds to the mRNA of p62 (Figure 3I). Finally, because CPEB1 regulates protein translation by promoting or inhibiting poly(A) elongation 8, we performed a poly(A) tail length assay followed by Sanger sequencing, which showed that CPEB1 attenuated poly(A) tail elongation at the 3'-UTR of *p62* mRNA (Figure 3J). Taken together, these data suggest that CPEB1 loss promotes p62 translation by facilitating poly(A) elongation of its mRNA.

### CPEB1-p62-KEAP1 axis controls NRF2 proteostasis and ferroptosis susceptibility in cancer

**Figure 4:**
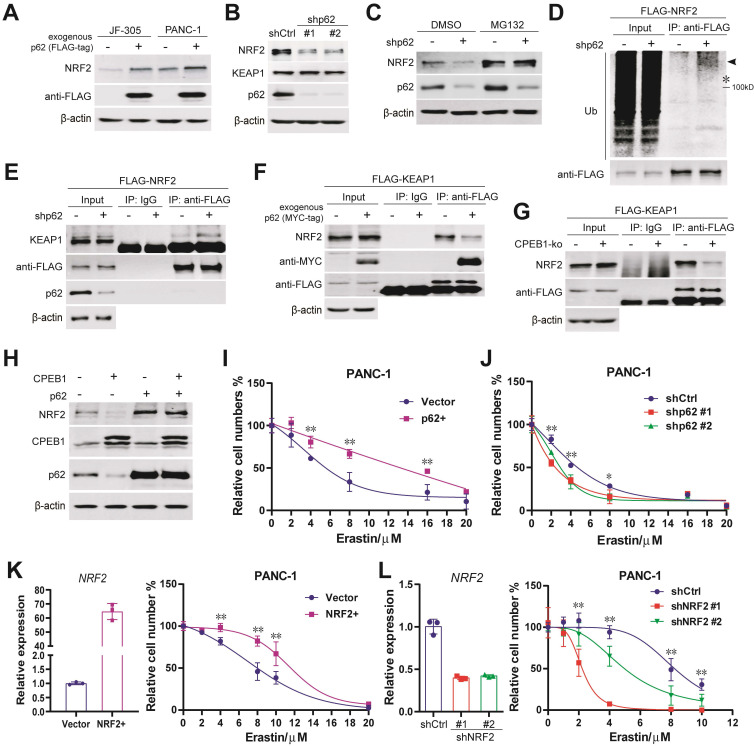
** CPEB1-p62-KEAP1 axis controls NRF2 proteostasis and ferroptosis susceptibility in pancreatic cancer. (A)**. The abundance of NRF2 in PANC-1 and JF-305 cells, without or with exogenous FLAG-tagged p62 overexpression, was detected by immunoblotting. **(B)**. The abundance of NRF2 and KEAP1 in PANC-1 cells, without (shCtrl) or with p62 knockdown (shp62), was determined by immunoblotting. **(C)**. PANC-1 cells, without or with p62 knockdown, were treated with DMSO or MG132 (10 μM for 12 hours), and the abundance of NRF2 was determined by immunoblotting. **(D)**. PANC-1 cells expressing exogenous FLAG-tagged NRF2 (FLAG-NRF2), without or with p62 knockdown, were used for anti-FLAG immunoprecipitation and immunoblot analysis, pretreated with MG132 of 10 μM for 12 hours. *represents the band location of FLAG-tagged NRF2. **(E)**. Cell lines and pretreatment used in (D) were used for anti-FLAG co-immunoprecipitation and immunoblots. **(F-G)**. PANC-1 cells expressing exogenous FLAG-tagged KEAP1 (FLAG-KEAP1), (F) without or with p62 (MYC-tag) overexpression or (G) without or with CPEB1 knockout, were used for anti-FLAG co-immunoprecipitation and immunoblots. **(H)**. Exogenous CPEB1 was expressed or not in the PANC-1 derivative cells (without or with p62 overexpression), and the abundance of NRF2 was determined by immunoblotting. **(I-J)**. Derivatives of (I) PANC-1 cells without (Vector) or with exogenous p62 overexpression (p62+) and (J) PANC1 cells without (shCtrl) or with p62 knockdown (shp62) were treated with indicated doses of erastin for 72 hours, and cell numbers were determined by CCK-8 assay. *P < 0.05; **P < 0.01. **(K-L)**. Derivatives of (K) PANC-1 cells without (Vector) or with exogenous NRF2 overexpression (NRF2+) and (L) PANC-1 cells without (shCtrl) or with NRF2 knockdown (shNRF2) were treated with erastin of indicated doses for 72 hours, and cell numbers were determined by CCK-8 assay. The overexpression (K) or knockdown (L) of NRF2 was validated using RT-qPCR as shown in left panel, and CCK-8 data was shown in right panel. *P < 0.05; **P < 0.01.

The upregulation of p62 and NRF2 in CPEB1 deficient cells led us to hypothesize that the CPEB1-p62-KEAP1 axis regulates NRF2 proteostasis and ferroptosis in pancreatic cancer. A number of complementary experiments were conducted to test this hypothesis. First, exogenous expression of p62 resulted in an increased abundance of NRF2 in JF-305 and PANC-1 cells (Figure 4A); conversely, knockdown of p62 induced a downregulation of NRF2 abundance and the transcriptional repression of NRF2-target genes (Figure 4B, Figure S7A), without affecting NRF2 transcription and KEAP1 abundance (Figure S7B, Figure 4B). Second, the decrease in NRF2 abundance induced by p62 knockdown was reversed by the proteasome inhibitor MG132 (Figure 4C). Third, the level of poly-ubiquitinated NRF2 was remarkably increased in response to p62 knockdown (Figure 4D). Fourth, the interaction between KEAP1 and NRF2 was significantly enhanced by p62 knockdown (Figure 4E) and attenuated by either p62 overexpression (Figure 4F) or CPEB1 deletion (Figure 4G). Finally, overexpression of exogenous p62 prevented CPEB1-induced NRF2 degradation (Figure 4H), supporting the critical role of p62 in mediating the CPEB1-controled regulation of NRF2 stability.

To validate the role of this axis in controlling ferroptosis susceptibility, we demonstrated that overexpression or knockdown of p62 remarkably improved or reduced cellular resistance to erastin, respectively, by CCK-8 assays (Figure 4I-J). The expected key role of NRF2 in mediating the cellular response to erastin in pancreatic cancer cells was also confirmed using NRF2 overexpressing or knockdown cell models (Figure 4K-L). All these results support the notion that the CPEB1-p62-KEAP1 axis functions as a critical regulatory pathway for controlling NRF2 homeostasis and susceptibility to ferroptosis in pancreatic cancer.

To further assess the ubiquity of the axis in cancer, we overexpressed CPEB1 in multiple cancer cell lines and confirmed that p62 and NRF2 were downregulated in pancreatic and colorectal cancer (Figure S8A-B), while no change was observed in gastric cancer (Figure S8C). Additionally, we demonstrated TWIST1 was the downstream ferroptosis-regulator of CPEB1 in gastric cancer as reported (Figure S8D) 25.

### CPEB1-deficient tumor is resistant to erastin therapy *in vivo*

**Figure 5:**
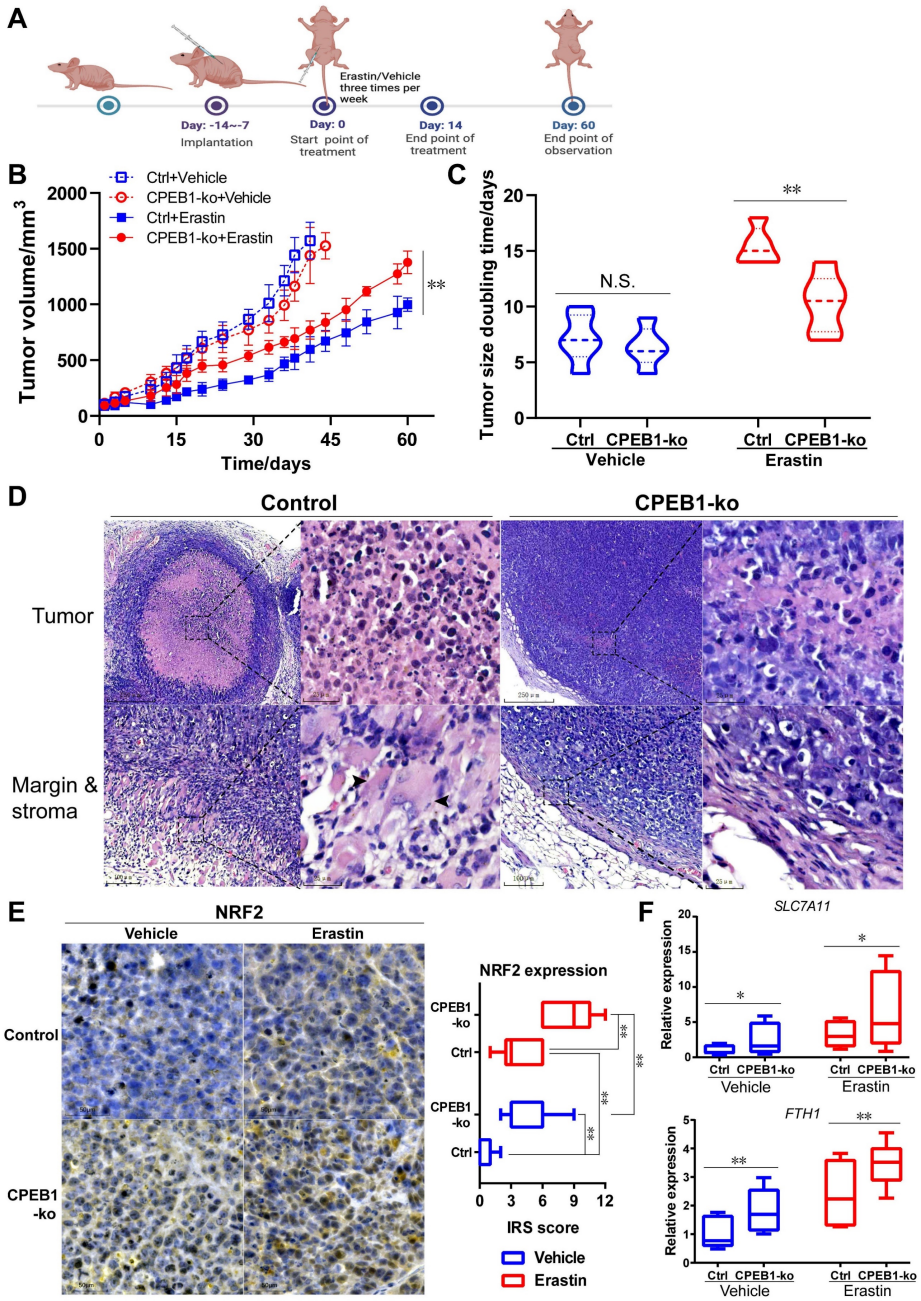
** CPEB1-deficient pancreatic cancer shows resistant to erastin treatment *in vivo.* (A)**. The treatment schedule of erastin in nude mice. **(B).** Tumor growth curve of each group following treatment of vehicle or erastin. The mice numbers in each group at the end point of observation: Ctrl+Vehicle, n=6; CPEB1-ko+Vehicle, n=5; Ctrl+Erastin, n=5; CPEB1-ko+Erastin, n=6. **(C).** The doubling time of tumor size in each group following the treatment of vehicle or erastin. **(D)**. Representative xenograft tumor sections with H&E staining of JF-305 derived CPEB1-normal (Control) and CPEB1-deficient (CPEB1-ko) tumors. Remarkable cell death displaying cell body expansion, membrane damage and relatively central-placed, intact and normal-sized nuclei was observed in CPEB1-normal tumors (upper panels in Control, x100 and x400 magnification, respectively), with remarkable tumor shrinking accompanied by surrounding granulomatous reaction (bottom panels in Control, x200 and x400 magnification, respectively; arrows show multinucleated giant cells) at tumor edge; in contrast, sparsely small focal cell death was observed in CPEB1-deficient tumors (upper panels in CPEB1-ko, x100 and x400 magnification, respectively), without tumor shrinking and surrounding granulomatous reaction (bottom panels in CPEB1-ko, x200 and x400 magnification, respectively). **(E)**. Representative sections with IHC staining of NRF2 using the above tumor tissue (left panels, x200 magnification), and the quantification of NRF2 expression was performed using immunoreactive score (IRS) (right panel). **(F)**. Total RNA from the above tumor tissue (D) were extracted by three samples per tumor (3 tumors per group), used for RT-qPCR assay with indicated primers. Relative expression level of indicated genes was evaluated using 2-ΔCT, normalized with the mean value of the first group from left in each panel. *P<0.05; **P < 0.01; N.S., no significance.

Encouraged by the above *in vitro* results, to further evaluate the effect of CPEB1 loss on ferroptosis susceptibility *in vivo*, we performed subcutaneous xenograft tumor models derived from JF-305 cells without or with CPEB1 knockout in nude mice, and treated the mice with erastin or vehicle, as shown in Figure 5A. We demonstrated that CPEB1-deficient tumors conferred remarkable resistance to erastin treatment, with less inhibition of tumor growth (Figure 5B) and a shorter doubling time in tumor size (Figure 5C), compared to their CPEB1-normal counterparts.

To better understand the differential susceptibility to ferroptosis inducer caused by CPEB1 loss, we evaluated the histological response of xenograft tumors to erastin or vehicle treatment using pathological slides. In the vehicle-treated group, no morphological difference was observed between CPEB1-normal and -deficient tumors on hematoxylin-eosin (H&E) stained slides (Figure S9A); whereas in the erastin-treated group, massive cell death was observed in CPEB1-normal tumors (Figure 5D, left panels), characterized by cell swelling, membrane damage and relatively normal-sized nuclei without pyknosis or karyorrhexis, accompanied by a remarkable tumor shrinkage and surrounding granulomatous reaction at the tumor edge; in contrast, only sparsely small focal cell death was observed in CPEB1-deficient tumors, without tumor shrinkage and granulomatous reaction (Figure 5D, right panels). Consistent with *in vitro* results, a significantly higher expression of NRF2 was observed in CPEB1-deficient tumors than in CPEB1-normal tumors in both vehicle- and erastin-treated groups, as determined by IHC assay (Figure 5E), using CPEB1 as a positive control (Figure S9B). Similarly, CPEB1-deficient tumors displayed higher p62 expression (Figure S9C). We further examined the transcription of the NRF2-target anti-ferroptosis genes in the tumors after treatment with erastin or vehicle using RT-qPCR assay (Figure 5F), and confirmed that CPEB1-deficient tumors displayed a higher expression of *SLC7A11* and* FTH1* compared to CPEB1-normal tumors, consistent with the ferroptosis-resistant phenotype. All these results suggest that CPEB1-deficient tumors are ferroptosis-resistant, which led us to explore its clinical role.

### The expression of CPEB1, p62 and NRF2 in pancreatic cancer and their prognostic significance

**Figure 6:**
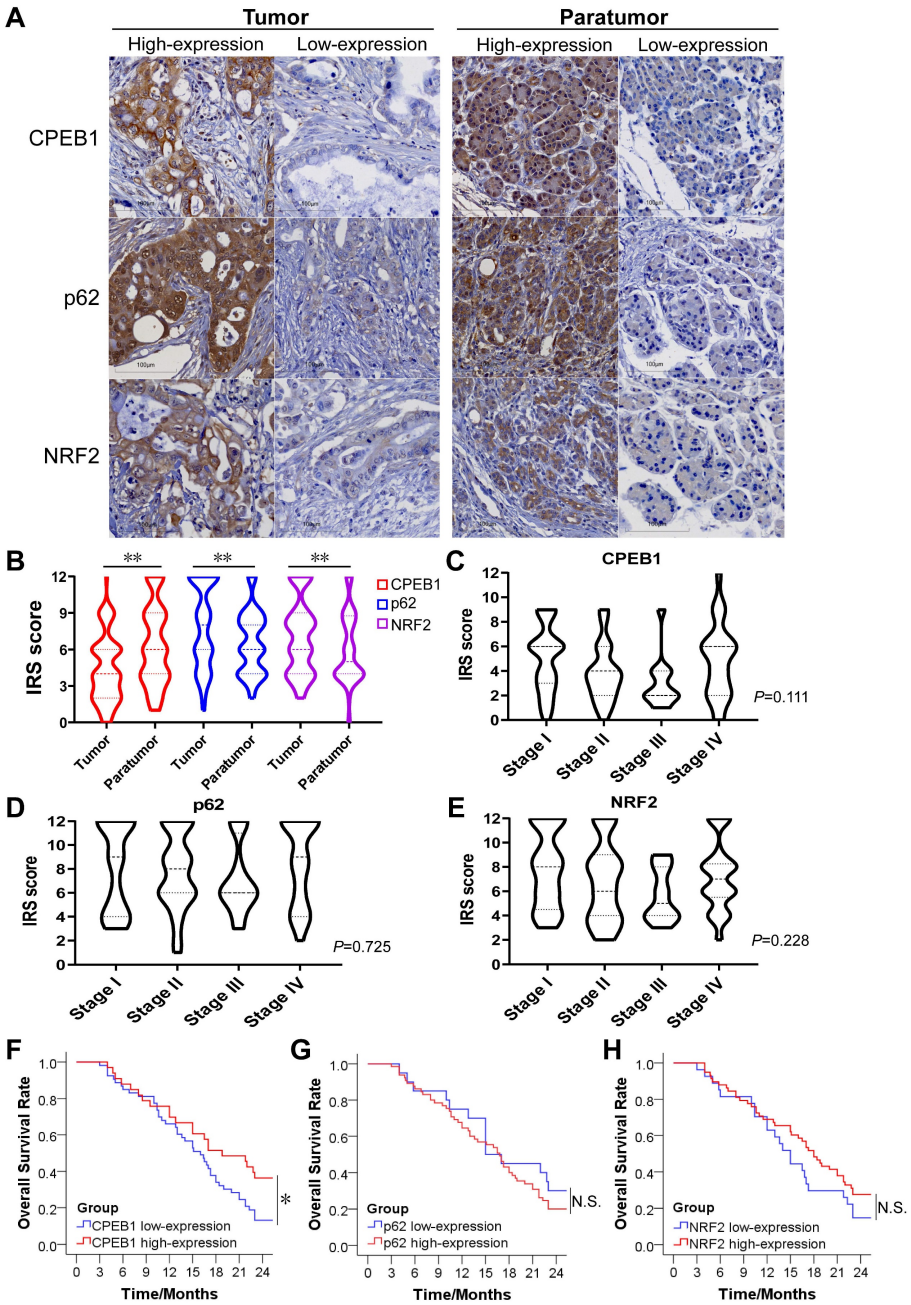
** The expression of CPEB1, p62 and NRF2 in pancreatic cancer and their clinical significance. (A)**. Representative sections with immunohistochemical staining of CPEB1, p62 and NRF2 in pancreatic cancer (left panels) and paratumor tissue (right panels). **(B)**. Expression level of CPEB1, p62 and NRF2 analyzed using IRS between tumor and paratumor tissue. **(C-E)**. Expression of CPEB1 (C), p62 (D) and NRF2 (E) in different TNM stage of pancreatic cancer. **(F-H)**. The 2-year overall survival of the pancreatic cancer patients with high- or low- expression of CPEB1 (F), p62 (G) and NRF2 (H) in tumor tissue. *P < 0.05; **P < 0.01; N.S., no significance.

To evaluate the clinical significance of CPEB1, p62 and NRF2 in pancreatic cancer, we collected tissue samples and clinical information from 90 patients with confirmed pancreatic ductal adenocarcinoma who underwent surgical resection. The basic characteristics of the included patients were summarized in Table S1. The expression of CPEB1, p62 and NRF2 in tumor and tumor-adjacent tissues was evaluated by IHC assay (Figure 6A). We found that the expression level of CPEB1 was significantly lower in the tumor tissues than in the adjacent tissues, whereas the expression levels of NRF2 and p62 were higher in the tumor tissues than in the adjacent tissues (Figure 6B). Within the tumor tissue, the expression of CPEB1, p62 and NRF2 was independent of pathological TNM stage (Figure 6C-E). The relationship between clinicopathological parameters and the expression of CPEB1, p62 and NRF2 is shown in Table 1. Low-expression of CPEB1 was associated with later T stage (*P*<0.05), but not with N and M stage and other clinical factors. Interestingly, the expression of CPEB1 was negatively correlated with the expression of p62 in the tumor (R=-0.241, *P*<0.05), but was not correlated with the expression of NRF2 (Table 2).

The 2-year overall survival (OS) rate for all patients was 23.3%, with a median survival time of 16.7 months after surgery. Patients with CPEB1 high-expression tumors had a significantly higher 2 year-OS rate than those with CPEB1 low-expression tumors (36.4% vs. 13.2%, *P*=0.034) (Figure 6F), whereas no significant difference in survival was observed between the p62 high- and low-expression groups (Figure 6G), nor between NRF2 high- and low-expression groups (Figure 6H).

To identify independent prognostic factors for survival, clinicopathological parameters including gender, age, tumor location, histological differentiation, pathological TNM stage, vascular invasion, neural invasion, serum CEA, CA199, and CPEB1 expression were analyzed using the Cox proportional hazards regression model. Multivariate analysis showed that CPEB1, pathological TNM stage and serum CEA level were independent prognostic factors for OS (Table 3). Hence, our results support the notion that CPEB1 is a potential prognosticator in pancreatic cancer.

## Discussion

Due to poor prognosis and limited response to chemotherapy, targeting ferroptosis is becoming a promising option for pancreatic cancer patients, particularly for those with metastatic and chemoresistant tumors 4, 5. In this study, we discovered CPEB1 as a key regulator controlling NRF2 proteostasis and ferroptosis susceptibility in pancreatic cancer, and established the clinical significance of CPEB1 in predicting therapeutic outcomes.

Ferroptosis therapy for pancreatic cancer has been rapidly developed in recent years. Statins, as the applicable ferroptosis inducers, have displayed encouraging therapeutic effects in pancreatic cancer: two prospective clinical trials (NCT01124786 and NCT00844649) have demonstrated a significantly prolonged overall survival and progression-free survival brought by statins to late-stage pancreatic cancer patients 33, whereas another non-statin ferroptosis inducer Sorafenib failed to provide a remarkable survival benefit 34, 35. As an increasing number of ferroptosis reagents are entering clinical trials or seeking approval in recent years, it is essential to identify appropriate patients who may benefit from ferroptosis therapy. Our study contributes to the stratification of patients with pancreatic cancer for ferroptosis therapy by providing a feasible biomarker.

As a group of pivotal translation regulators, the CPEB family consists of four homologues 7, with CPEB1 and CPEB4 receiving the most attention in cancer research 36. Despite having similar functional domains and RNA affinities for targeting the CPE sequence in the 3'-UTR of mRNA to facilitate or inhibit translation 7, 36, the oncological significance of CPEB1 and CPEB4 is completely opposite: CPEB4 promotes tumor proliferation, migration, invasion, and vascularization 36, 37, whereas CPEB1 inhibits cancer progression as a tumor suppressor 14, 38. In contrast to CPEB4, whose oncoprotein function in pancreatic cancer has been identified 36, the biological function and clinical significance of CPEB1 in pancreatic cancer are yet to be elucidated. In addition, there are limited data on the role of CPEB1 in ferroptosis, just one study reported that CPEB1 promotes ferroptosis susceptibility in gastric cancer by suppressing TWIST1 expression 25, which does not apply to pancreatic cancer as indicated above. The significance of our findings lies in two aspects: first, we identified CPEB1 as a ferroptosis modulator critical for therapeutic response in pancreatic cancer; and second, we revealed that p62 and NRF2 are essential downstream targets of CPEB1, mediating the regulation of ferroptosis susceptibility. Notably, the p62-NRF2 cascade plays a crucial but not exclusive role in the CPEB1-mediated ferroptosis regulation, supported by the proteomic data that there are numerous downstream ferroptosis-associated genes of CPEB1 that are not targeted by NRF2, highlighting the complexity of CPEB1's regulatory mechanism for ferroptosis.

Different with other regulators of ferroptosis, CPEB1 modulates cellular ferroptosis through translation rather than directly regulating enzyme activity, transcription, or protein degradation. The mechanism by which CPEB1 regulates ferroptosis is novel and distinctive, as translational regulation in ferroptosis has been largely unknown 5, 6. Moreover, CPEB1 is closely associated with the prognosis of malignant tumors in clinic, as demonstrated by our study and published open data, suggesting that it has the potential to be developed into a clinical biomarker or a therapeutic target.

As a downstream effector of CPEB1, NRF2 is a critical modulator of oxidative stress, iron metabolism and ferroptosis 26, 39, 40. Generally, upregulated or activated NRF2 promotes iron utilization and antioxidant responses in cancer cells, inducing cellular resistance to ferroptosis by activating the transcription of anti-ferroptosis genes 27, 40, 41. A typical example of the target gene is *GPX4*, which plays a key role in protecting cells from oxidative damage by preventing membrane lipid peroxidation 42, 43. Another target gene, *HMOX1*, is responsible for catabolism of cytosolic heme to ferrous iron and biliverdin, thereby facilitating the release and utilization of ferrous iron 42. It is well established that NRF2 proteostasis is controlled by the p62-KEAP1 interaction, within which KEAP1 functions as an E3 ubiquitin ligase by targeting NRF2 for poly-ubiquitination and proteasome-dependent degradation 30, 39_;_ whereas p62 enhances NRF2 stability by abrogating the KEAP1-mediated NRF2 degradation 30, 31. Thus, CPEB1 loss-induced p62 upregulation remarkably improves NRF2 proteostasis, which benefits cancer cells from antioxidative stress and ferroptosis resistance.

In addition to interacting with KEAP1, p62 is also an important adaptor protein in autophagy and a signaling hub that mediates multiple cellular functions, such as activation of mTORC1 and NF-κB 44. Accumulating evidence suggests that p62 is an important oncoprotein 45-47, particularly in pancreatic cancer, where p62 promotes tumor progression by stabilizing NRF2, which modulates the stress response and malignant phenotype 46. Despite the importance of p62 in cancer, its translational regulation remains unclear as most studies have focused on its degradation by ubiquitination or autophagy. Our study revealed that CPEB1 is a p62 suppressor that targets protein translation, highlighting the significant function of CPEB1 as a tumor-suppressive translational regulator.

Due to the limited sample size of this study, the prognostic value of CPEB1 remains to be further validated by more convincing clinical evidence. However, based on our finding, detecting expression level of CPEB1 in pancreatic cancer is potentially informative for the clinical decisions when ferroptosis therapy is used in clinic.

## Supplementary Material

Supplementary figures and tables.

## Figures and Tables

**Table 1:** The association between clinicopathological parameters and the expression of CPEB1/p62/NRF2

	CPEB1 expression		*P* value	p62 expression		*P* value	NRF2 expression		*P* value
	Low(%)	High(%)		Low(%)	High(%)		Low(%)	High(%)	
**Gender**									
Male	30(56.6)	20(60.6)	0.714	13(65)	36(55.4)	0.447	11(40.7)	38(65.5)	0.031
Female	23(43.4)	13(39.4)		7(35)	29(44.6)		16(59.3)	20(34.5)	
**Age**									
<58.5	27(50.9)	16(48.5)	0.825	11(55)	32(49.2)	0.652	15(55.6)	27(46.6)	0.44
≥58.5	26(49.1)	17(51.5)		9(45)	33(50.8)		12(44.4)	31(53.4)	
**Tumor location**									
Head	40(75.5)	24(72.7)	0.777	14(70)	49(75.4)	0.631	21(77.8)	42(72.4)	0.599
Body and tail	13(24.5)	9(27.3)		6(30)	16(24.6)		6(22.2)	16(27.6)	
**Histopathological grade**									
Ⅰ-Ⅱ	37(69.8)	21(63.6)	0.552	15(75)	42(64.6)	0.388	21(77.8)	37(63.8)	0.197
Ⅲ	16(30.2)	12(36.4)		5(25)	23(35.4)		6(22.2)	21(36.2)	
**T stage**									
T1-2	21(39.6)	22(66.7)	0.047	13(65)	29(44.6)	0.267	14(51.9)	29(50)	0.771
T3	24(45.3)	9(27.3)		5(25)	28(43.1)		9(33.3)	23(39.7)	
T4	8(15.1)	2(6.1)		2(10)	8(12.3)		4(14.8)	6(10.3)	
**N stage**									
N0	17(32.1)	15(45.5)	0.365	8(40)	24(36.9)	0.603	6(22.2)	26(44.8)	0.12
N1	29(54.7)	13(39.4)		8(40)	33(50.8)		16(59.3)	25(43.1)	
N2	7(13.2)	5(15.2)		4(20)	8(12.3)		5(18.5)	7(12.1)	
**M stage**									
M0	42(79.2)	21(63.6)	0.112	14(70)	48(73.8)	0.735	22(81.5)	41(70.7)	0.29
M1	11(20.8)	12(36.4)		6(30)	17(26.2)		5(18.5)	17(29.3)	
**Vascular invasion**									
No	30(56.6)	21(63.6)	0.519	14(70)	36(55.4)	0.245	17(63)	34(58.6)	0.704
Yes	23(43.4)	12(36.4)		6(30)	29(44.6)		10(37)	24(41.4)	
**Neural invasion**									
No	18(34)	13(39.4)	0.61	10(50)	21(32.3)	0.151	11(40.7)	20(34.5)	0.577
Yes	35(66)	20(60.6)		10(50)	44(67.7)		16(59.3)	38(65.5)	
**Serum CEA (ng/ml)**									
≤5	38(76)	19(61.3)	0.159	13(76.5)	43(68.3)	0.512	14(53.8)	41(75.9)	0.046
>5	12(24)	12(38.7)		4(23.5)	20(31.7)		12(46.2)	13(24.1)	
**Serum CA199 (U/ml)**									
≤37	11(21.6)	7(22.6)	0.915	0(0)	17(26.6)	0.04	4(15.4)	13(23.6)	0.395
>37	40(78.4)	24(77.4)		17(100)	47(73.4)		22(84.6)	42(76.4)	

**Table 2:** The association between the expression of p62, NRF2 and CPEB1

	CPEB1 expression		R value	*P* value
	Low(%)	High(%)		
**p62 expression**				
Low	8(15.4)	12(36.4)	-0.241	0.026
High	44(84.6)	21(63.6)		
**NRF2 expression**				
Low	19(37.3)	7(21.2)	0.169	0.123
High	32(62.7)	26(78.8)		

**Table 3:** Multivariate analysis of overall survival by Cox proportional hazards regression

Variable	HR	95%CI	P value
**CPEB1**			
Low-expression	1		
High-expression	0.428	0.345-0.94	0.007
**TNM stage**			<0.001
Ⅰ	1		
Ⅱ	4.484	1.653-12.164	0.003
Ⅲ	17.922	4.877-65.862	<0.001
Ⅳ	60.701	17.716-207.986	<0.001
**Serum CEA (ng/ml)**			
≤5	1		
>5	2.167	1.137-4.131	0.019
**Histopathological grade**			0.125
Ⅰ	1		
Ⅱ	3.105	1.044-9.237	0.042
Ⅲ	1.329	0.712-2.482	0.371
**Gender**			
Male	1		
Female	1.801	0.99-3.277	0.054
**Age**			
<58.5	1		
≥58.5	1.115	0.633-1.964	0.707
**Tumor location**			
Head	1		
Body and tail	0.658	0.337-1.287	0.222
**Vascular invasion**			
No	1		
Yes	1.394	0.687-2.83	0.358
**Neural invasion**			
No	1		
Yes	1.654	0.792-3.454	1.181
**Serum CA199 (U/ml)**			
≤37	1		
>37	0.808	0.404-1.615	0.546

## Abbreviations

ATCC: american type culture collection
CCK-8: cell counting kit-8
cDNA: complementary DNA
CPEB1: cytoplasmic polyadenylation element binding protein 1
CPEs: cytoplasmic polyadenylation elements
DEPs: differentially expressed proteins
DCFH-DA: 2',7'-dichlorodihydrofluorescein diacetate
DIA: data-independent acquisition
DMEM: dulbecco's modified eagle's medium
ECACC: european collection of authenticated cell cultures
ePAT: extension poly(A) test
FBS: fetal bovine serum
GSH: glutathione
GSSG: glutathione disulfide
H&E: hematoxylin-eosin
IHC: Iimmunohistochemistry
IRS: Iimmunoreactive score
KEGG: kyoto encyclopedia of genes and genomes
LDH: lactate dehydrogenase
MDA: malondialdehyde
MOI: multiplicity of infection
mTORC1: mammalian target of rapamycin complex 1
NF-κB: nuclear factor κB
NRF2: nuclear factor erythroid 2-related factor 2
OS: overall surviva
PDAC: pancreatic ductal adenocarcinoma
PVDF: polyvinylidene fluoride
RIP: ribonucleoprotein immunoprecipitation
ROS: reactive oxygen species
RPMI: roswell park memorial Institute
RT-qPCR: quantitative real-time PCR
SDS: sodium dodecyl sulfate
siRNA: small interfering RNA
TCGA: the cancer genome atlas
TNM: tumor-node-metastasis
UTR: untranslated region
