# Supplementary material for: CPEB1 Controls NRF2 Proteostasis and Ferroptosis Susceptibility in Pancreatic Cancer
Source: Int J Biol Sci. 2024 Jun 3;20(8):3156–72. doi: 10.7150/ijbs.95962 (PMC11186365; doi:10.7150/ijbs.95962)
Supplement: Supplementary file 1 — Supplementary figures and tables. [file ijbsv20p3156s1.pdf]

Supplementary Figures

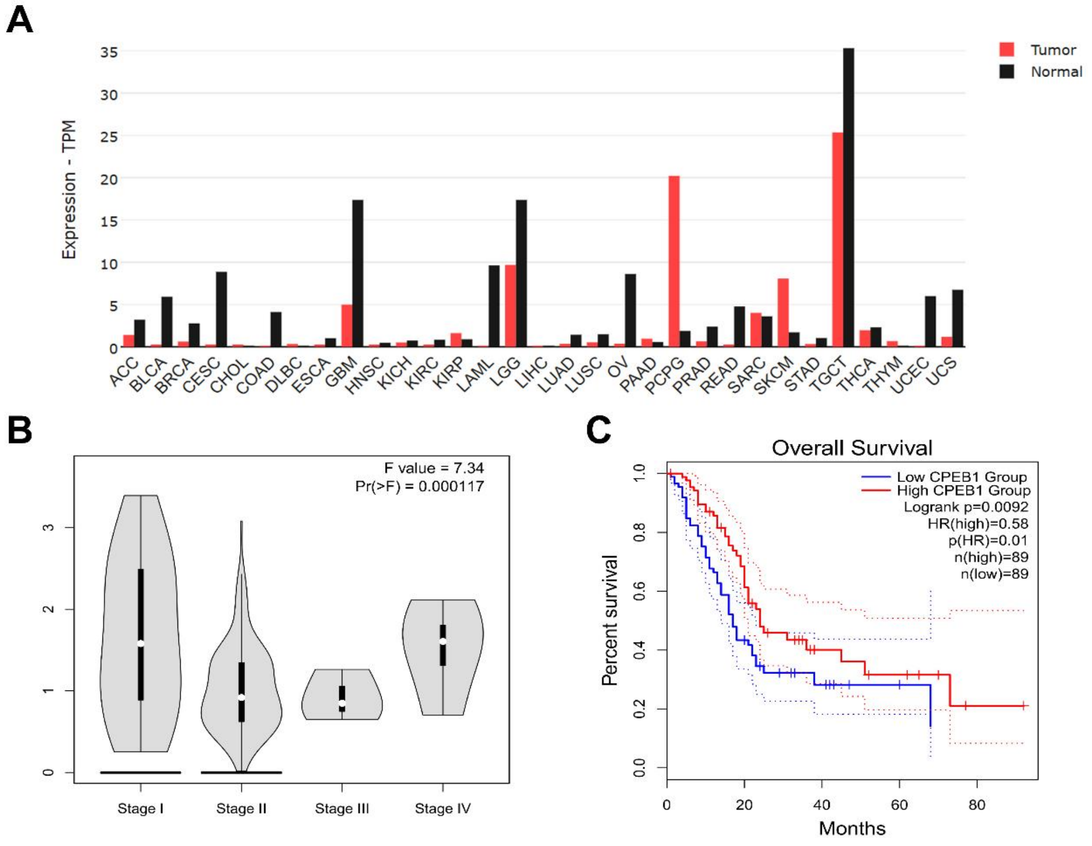

Figure S1

(A). The expression of CPEB1 in multiple cancer and paratumor tissue was analyzed using TCGA data based on RNA-seq database, via the GEPIA portal (*Tang, Z. et al. Nucleic Acids Res 2017, 10.1093/nar/gkx247*; http://gepia.cancer-pku.cn/detail.php).

(B). The expression level of CPEB1 in different TNM stage in pancreatic cancer from TCGA data was analyzed using the "Stage Plot" model of the GEPIA portal.

(C). The Kaplan-Meier survival analysis was performed for the pancreatic cancer patients with high- or low- expression of CPEB1, using the data from TCGA via the GEPIA portal.

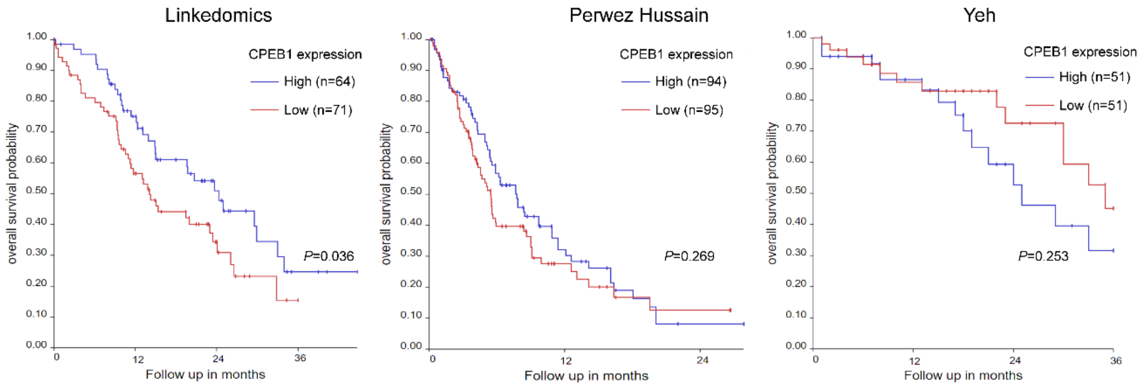

Figure S2

The association of CPEB1 expression and overall survival in pancreatic cancer based on Kaplan-Meier survival analysis in three independent cohorts, using the R2: Genomics Analysis and Visualization Platform (*http://r2.amc.nl*).

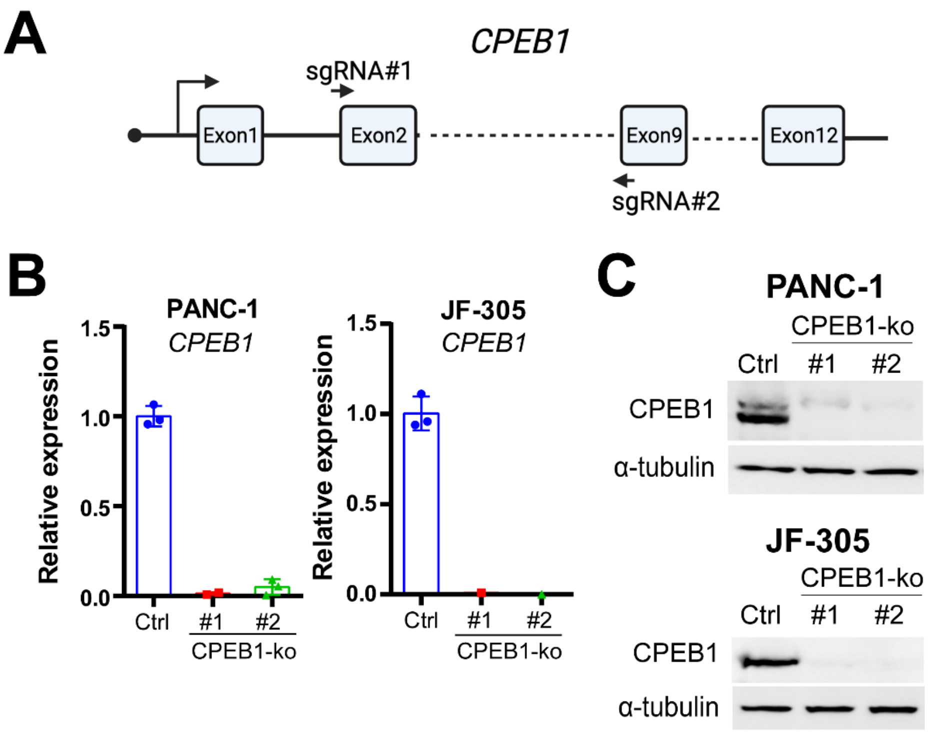

Figure S3

(A). The design of sgRNA sequences for CPEB1 knockout using CRISPR-Cas9 system.

(B-C). RT-qPCR (B) and immunoblots (C) were performed to validate *CPEB1* knockout outcome. Two clones of knockout cells (#1 and #2) were examined for each cell line. Arrow shows the CPEB1 protein in panel C.

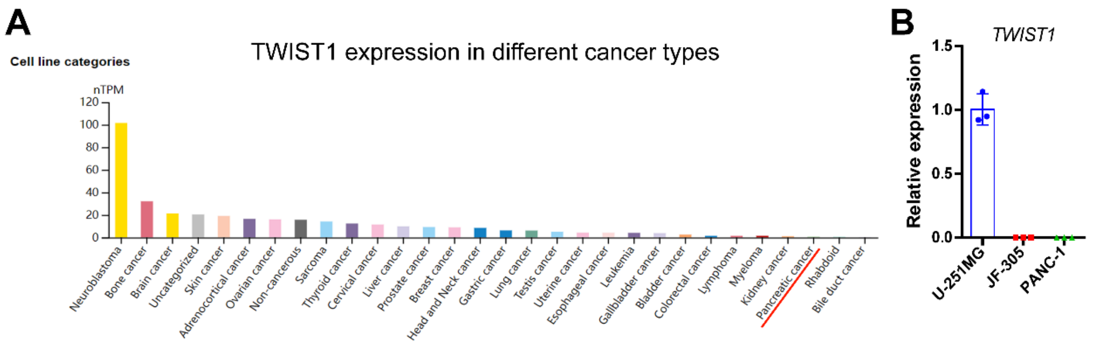

Figure S4

(A). The expression of TWIST1 in multiple cancer cell lines was analyzed using The Human Protein Atlas database (*Uhlén M, et al. Science 2015, 347(6220):1260419*; *https://www.proteinatlas.org/*). Pancreatic cancer is labeled with red line.

(B). The mRNA level of TWIST1 in the JF-305 and PANC-1 cells was determined by RT-qPCR assay, using *TWIST1* expressing cell line U-251MG (glioblastoma) as a control.

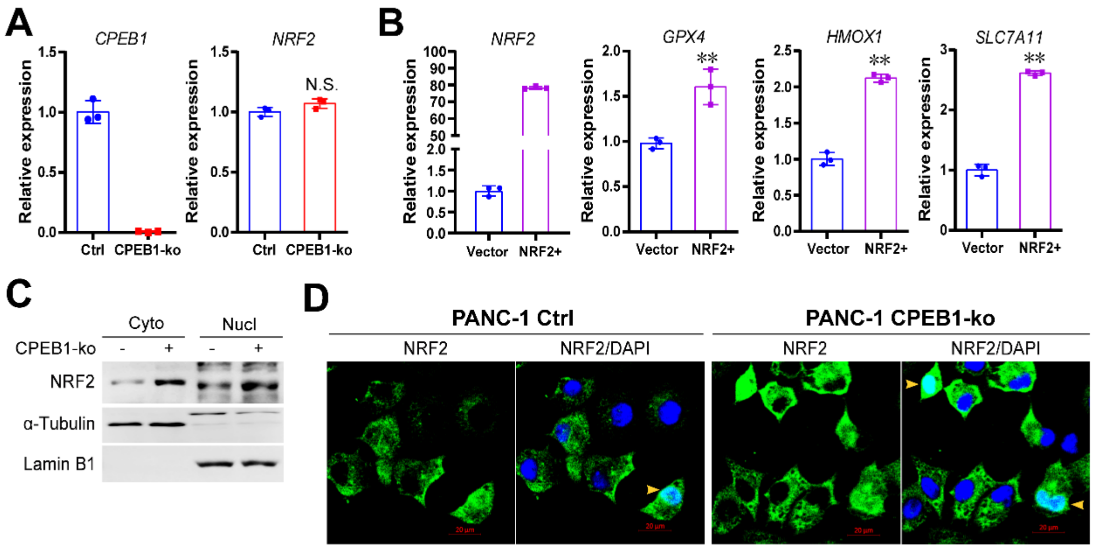

Figure S5

(A). The mRNA level of NRF2 in the PANC-1 cells without (Ctrl) or with CPEB1 knockout (CPEB1-ko) was determined using RT-qPCR assay.

(B). Total RNA from PANC-1 cells, without (Vector) or with NRF2 overexpression (NRF2+), were used for RT-qPCR assay using indicated primers.

(C). Cytoplasmic (Cyto) and nuclear (Nucl) proteins were isolated from the PANC-1 cells without (Ctrl) or with CPEB1 knockout (CPEB1-ko), and the abundance of NRF2 was determined by immunoblotting.

(D). Localization of NRF2 was detected by immunofluorescence assay (green color), merged with DAPI staining (blue color). Yellow arrows show nuclear localization of NRF2.

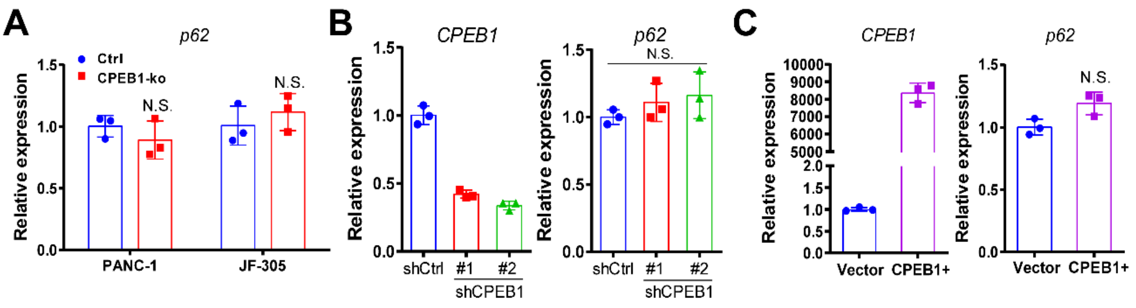

Figure S6

(A). The mRNA level of p62 in the PANC-1 and JF-305 derivative cells without (Ctrl) or with CPEB1 knockout (CPEB1-ko) was determined using RT-qPCR assay.

(B-C). The mRNA level of p62 in the PANC-1 cells without (shCtrl) or with CPEB1 knockdown (shCPEB1) (B), or without (Vector) or with CPEB1 overexpression (CPEB1+) (C) was determined using RT-qPCR assay.

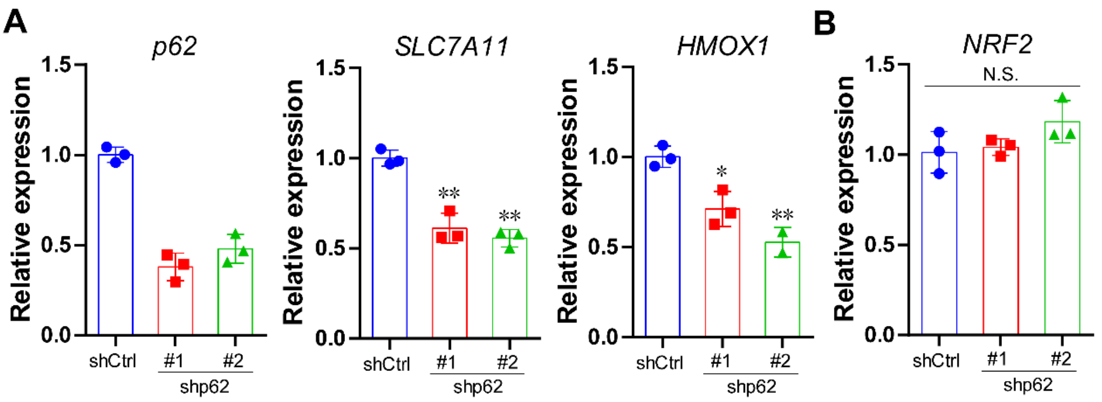

Figure S7

(A). Total RNA from PANC-1 cells, without (shCtrl) or with p62 knockdown (shp62), were used for RT-qPCR assay using indicated primers.

(B). The mRNA level of NRF2 in the PANC-1 cells without (shCtrl) or with p62 knockdown (shp62) was determined using RT-qPCR assay.

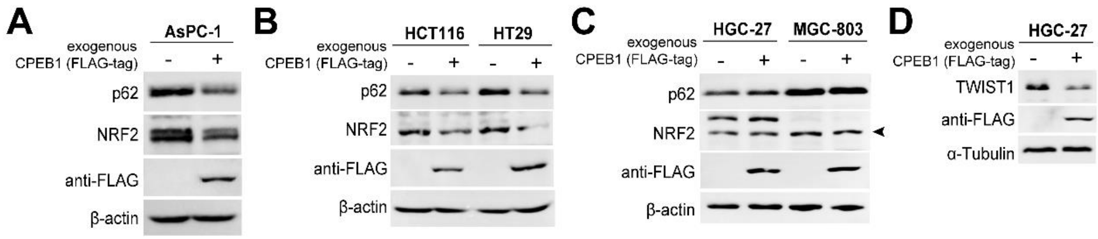

Figure S8

(A-C). The abundance of p62 and NRF2 in pancreatic cancer (AsPC-1) (A), colorectal cancer (HCT116 and HT29) (B) and gastric cancer (HGC-27 and MGC-803) cells (C), without or with exogenous CPEB1 (FLAG-tagged) overexpression, were determined by immunoblotting. Arrow shows the bands of NRF2.

(D). The abundance of TWIST1 in the HGC-27 cells without or with exogenous CPEB1 (FLAG-tagged) overexpression was determined by immunoblotting.

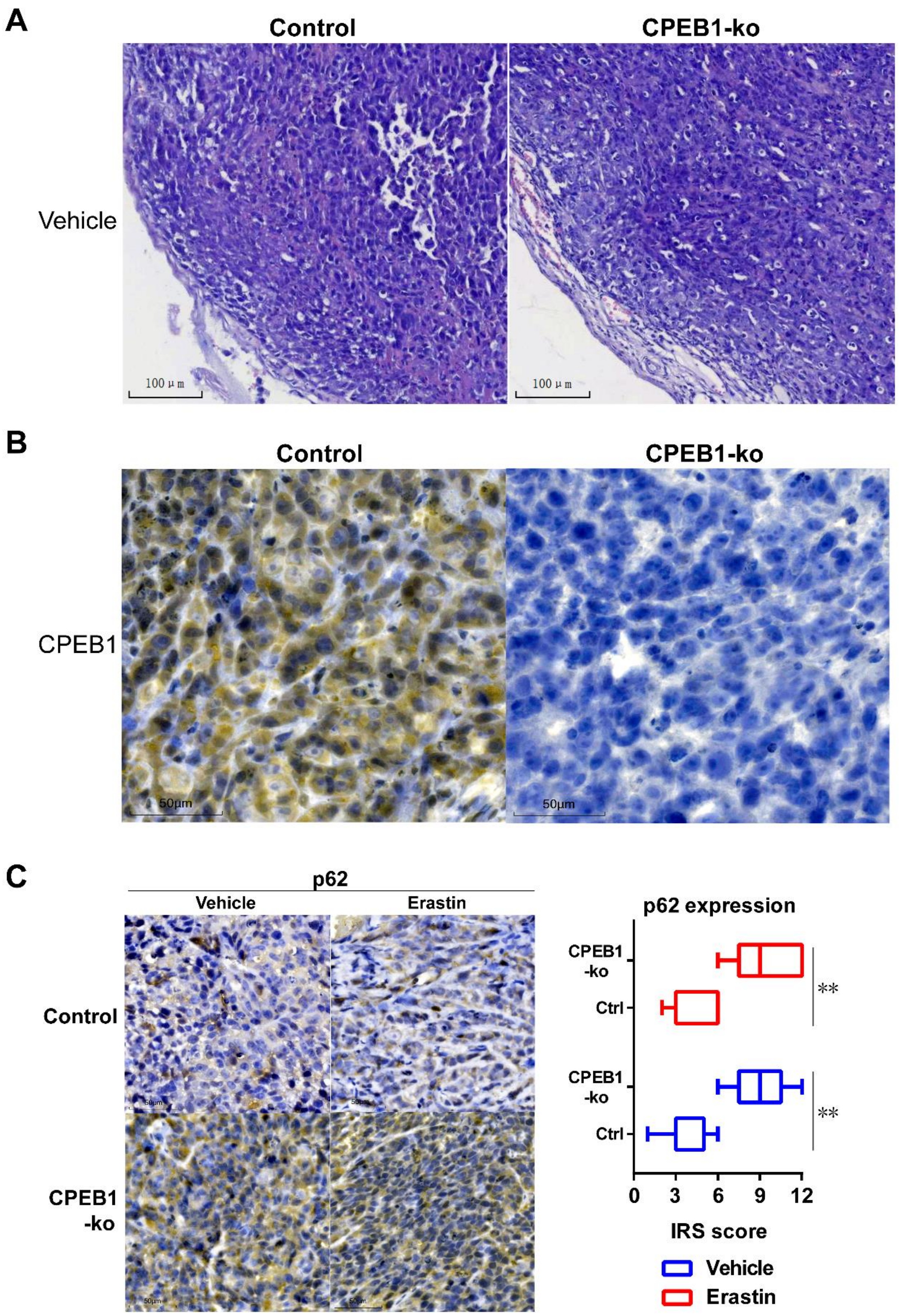

Figure S9

(A). Representative H&E sections of xenograft tumors derived from JF-305 without (left panel) or with CPEB1 knockout (right panel) treated with vehicle (corn oil), examined on the Day 9 from the start point of treatment (x200 magnification).

(B). Representative IHC sections of CPEB1 using the xenograft tumor tissue described in (A) (x400 magnification).
(C). Representative sections with IHC staining of p62 in the tumor tissue (left panels, x400 magnification), and the quantification of p62 expression using IRS (right panel).

Table S1. The baseline clinicopathological characteristics of the included patients

Clinicopathological parameters	No.(%)
**Median age**, years (range)	58.5 (33-77)
**Gender (M:F)**	53:37
**Tumor location**	
Head	67(74.4)
Body and tail	23(25.6)
**Type of surgery (%)**	
Whipple procedure	69(76.7)
Distal pancreatectomy	16(17.8)
Total pancreatectomy	5(5.5)
**Histopathological grade**	
I	6(6.7)
II	54(60)
III	30(33.3)
**TNM stage**	
I	24(26.7)
II	29(32.2)
III	13(14.4)
IV	24(26.7)
**Neural invasion**	
Yes	58(64.4)
No	32(35.6)
**Vascular invasion**	
Yes	37(41.1)
No	53(58.9)
**Serum CEA (ng/ml)**	
≤5	59(65.6)
>5	26(28.9)
Unknown	5(5.6)
**Serum CA199 (U/ml)**	
≤37	18(20)
>37	68(75.6)
Unknown	4(4.4)

Supplementary lists of materials

List 1. Primer information for qPCR

**Gene name**	**Forward (F) or Reverse (R)**	**Sequence (5' to 3')**
CPEB1	F	TGTCCTCCCAAAGGGTATGTG
	R	TGGGCTCCGGACAAAGTTAC
B2M	F	AGGCTATCCAGCGTACTCCA
	R	CTGCTTACATGTCTCGATCCCA
FTH1	F	TCCTACGTTTACCTGTCCATGT
	R	GTTTGTGCAGTTCCAGTAGTGA
GPX4	F	CGCGGGCTACAACGTCAAAT
	R	AGAGACGGTGTCCAAACTTGGTG
SLC7A11	F	GCGTGGGCATGTCTCTGAC
	R	GCTGGTAATGGACCAAAGACTTC
SQSTM1	F	GACTACGACTTGTGTAGCGTC
	R	AGTGTCCGTGTTTCACCTTCC
GAPDH	F	GGAGCGAGATCCCTCCAAAAT
	R	GGCTGTTGTCATACTTCTCATGG
NFE2L2	F	TCAGCGACGGAAAGAGTATGA
	R	CCACTGGTTTCTGACTGGATGT
HMOX1	F	AAGACTGCGTTCCTGCTCAAC
	R	AAAGCCCTACAGCAACTGTCG
TWIST1	F	GTCCGCAGTCTTACGAGGAG
	R	GCTTGAGGGTCTGAATCTTGCT

List 2. Primer information for poly(A) tail length assay

**Primer**	**Sequence (5' to 3')**
Anchor	GCGCGCTCCGCGGCCGCTTTTTTTTTTTT
ePAT-p62	CTTCCCGCAAGGCCTCTCAG

List 3. Reagents, kits and material information

**REAGENT or RESOURCE**	**SOURCE**	**IDENTIFIER**
**Antibodies**		
CPEB Antibody (E-10)	Santa Cruz Biotechnology	Cat#SC-137146
CPEB1 rabbit polyclonal antibody	Proteintech	Cat#13274-1-AP
Anti-CPEB1 polyclonal antibody	HUABIO	Cat#ER1902-92
SQSTM1 Polyclonal antibody	Proteintech	Cat#18420-1-AP
NRF2(D1Z9C)XP Rabbit mAb	Cell signaling technology	Cat#12721
NRF2 polyclonal antibody	Thermo Fisher Scientific	Cat#3PA5-27882
NRF2,NFE2L2 monoclonal antibody	Proteintech	Cat#66504-1-IG
Keap1 rabbit pAb	ABclonal	Cat#A21724
Anti-beta actin	Cell signaling technology	Cat#3700S
Anti-GPX4 Antibody	HUABIO	Cat#ET1706-45
xCT Rabbit mAb	Cell signaling technology	Cat#12691
Anti-Heme Oxygenase 1(HO-1) antibody	HUABIO	Cat#ET1604-45-50
Anti-Flag Tag	Sigma	Cat#F1804
BACH1 polyclonal antibody	Proteintech	Cat#14018-1-AP
FTH1 antibody	Cell signaling technology	Cat#3998
ubiquitin Polyclonal antibody	Proteintech	Cat#10201-2-AP
Alpha Tubulin Monoclonal antibody	Proteintech	Cat#66031-1-Ig
Fluorochrome-conjugated secondary antibody	Huabio	Cat# HA1121
Anti-Myc-tag Recombinant Mouse Monoclonal Antibody	HUABIO	Cat#HA60108
Peroxidase-Conjugated Goat anti-Rabbit IgG (H+L)	ZSGB-BIO	Cat#ZB-2301
Peroxidase-Conjugated Goat anti-Mouse IgG	ZSGB-BIO	Cat#ZB-2305

(H+L)		
**Chemicals and reagents**		
Cycloheximide	AbMole bioscience	Cat#M4879
DMEM cell culture medium	GIBCO	Cat#11995-065
RPMI 1640 cell culture medium	GIBCO	Cat#61870036
McCoy's 5A medium	GIBCO	Cat#16600082
Penicillin/streptomycin	Gibco	Cat#15140-122
Fetal bovine serum	Corning	Cat#35-010-CV
Corn oil	MedChemExpress	Cat#HY-Y1888
MG132	MedChemExpress	Cat#HY-13259
Chloroquine	MedChemExpress	Cat#HY-17589A
Erastin	MedChemExpress	Cat#HY-15763
RSL3	MedChemExpress	Cat#HY-100218A
DMSO	Sigma	Cat#67-68-5
TRIzol	TransGen Biotech	Cat#ET111-01-V2
Murine RNase inhibitor	Vazyme	Cat#R301-02
Klenow polymerase	New England Biolabs	Cat#M0210V
Protease inhibitor cocktail	Beyotime	Cat#P1005
DL2000 DNA Marker	TSINGKE	Cat#TSJ011
PageRuler	Thermo Fisher Scientific	Cat#26616
Glycogen	Beyotime	Cat#D0816
Sodium Deoxycholate	Sigma	Cat#D6750
Triethylammonium bicarbonate	Sigma	Cat#T7408
Dithiothreitol	Sigma	Cat#D9779
Lodoacetamide	Sigma	Cat#I1149
DAPI	Solarbia	Cat# C0065
Trypsin	Promega	Cat#V5111
**Cell Lines**		
293FT	Invitrogen	CAT#R700-07
PANC-1	ATCC	CAT#CRL-1469
HT29	ATCC	CAT#HTB-38
HCT116	ATCC	CAT#CCL-247
AsPC-1	ATCC	CAT#CRL-1682

U-251MG	ECACC	CAT#09063001
HGC-27	ECACC	CAT#94042256
MGC803	National Collection of Authenticated Cell Cuture (China)	CAT#TCHu 84
**Recombinant DNA**		
pSpCas9(BB)-2A-Puro (PX459)	Feng Zhang Lab; Nat Protoc. 8:2281	Addgene plasmid # 62988
pLKO.1	David Root Lab; Cell 124: 1283	Addgene plasmid #10878
CPEB1 (NM_030594) Human Tagged ORF Clone	ORIGEN	Cat#RC208662
**Critical Commercial Kits**		
Cell Counting Kit-8	Beyotime	Cat#C0039
MycoBlue Mycoplasma Detector	Vazyme	Cat#D101
GSSG/GSH Quantification Kit II	Dojindo	Cat#G263
LDH Assay Kit	Beyotime	Cat#C0016
MDA Assay Kit	Dojindo	Cat#M496
ROS Assay Kit	Beyotime	Cat#S0033S
BCA Protein Assay Kit	Beyotime	Cat#P0012S
TransStart Top Green qPCR SuperMix	TransGen Biotech	Cat#AQ132-21
WesternBright ECL	Advansta	Cat#K-12045-D50
Super ECL Detection Reagent ECL	Yeasen	Cat#36208ES76
TransStart® Top Green qPCR SuperMix (+Dye II)	TransGen Biotech	Cat# AQ132-21
SuperScript™ IV	Invitrogen	Cat#18090200
2×Phanta flash master mix	Vazyme	Cat#P510-01
Lipofectamine™ 2000	Invitrogen	Cat#11668030
FastPure Cell/T issue Total RNA isolution Kit V2	Vazyme	Cat#RC112-01
HiScript III 1st Strand cDNA Synthesis Kit (+gDNA wiper)	Vazyme	Cat#R312-02
SDS-PAGE Sample Loading Buffer，2X	Beyotime	Cat#P0015B

**Others**		
Immobilon-P PVDF membrane	Millipore	Cat#IPVH00010
0.45 μM NC membrane	Amershan protran	Cat#10600002
Pierce Protein A/G Agarose	Thermo Fisher Scientific	Cat#20421
anti-FLAG M2 agarose beads	Sigma	Cat#A2220
Klenow polymerase	New England Biolabs	Cat#M0210V
Tissue microarray slide	Outdo Biotech	Cat#HPanA170Su04

List 4. siRNA information

**Name**	**Sense or antisense**	**Sequence (5' to 3')**
siCPEB1#1	sense	GCUCUGCAUGGAAUGCUAATT
	antisense	UUAGCAUUCCAUGCAGAGCTT
siCPEB1#2	sense	GGUCUGACUUGGUGGACAATT
	antisense	UUGUCCACCAAGUCAGACCTT
Non-targeting control	sense	UUCUCCGAACGUGUCACGUdTdT
	antisense	ACGUGACACGUUCGGAGAAdTdT

List 5. shRNA sequence information

**Name**	**Sequence**
shNFE2L2#1	CCGGAGTTTGGGAGGAGCTATTATCCTCGAGGATAATAGCTCCTCCCAAACTTTTTG
shNFE2L2#2	CCGGGCTCCTACTGTGATGTGAAATCTCGAGATTTCACATCACAGTAGGAGCTTTTTG
shSQSTM1#1	CCGGCCTCTGGGCATTGAAGTTGATCTCGAGATCAACTTCAATGCCCAGAGGTTTTTG
shSQSTM1#2	CCGGCCGAATCTACATTAAAGAGAACTCGAGTTCTCTTTAATGTAGATTCGGTTTTTG
shCPEB1#1	CCGGCGTGTGACTTTCAATAACCAACTCGAGTTGGTTATTGAAAGTCACACGTTTTTG
shCPEB1#2	CCGGGCTCTGCATGGAATGCTAAATCTCGAGATTTAGCATTCCATGCAGAGCTTTTTG
Non-targeting control	CCGGGCGCGATAGCGCTAATAATTTCTCGAGAAATTATTAGCGCTATCGCGCTTTTTG

List 6. CPEB1-knockout sgRNA sequences

Control sgRNA	GTATCCTGACCTACGCGCTG
Exon 2 sgRNA	TGCACTACACCTATAAACCG
Exon 9 sgRNA	GATAAGCACAAGTATCCCAT
